# Marine Antitumor Drugs: Status, Shortfalls and Strategies

**DOI:** 10.3390/md8102702

**Published:** 2010-10-15

**Authors:** Ira Bhatnagar, Se-Kwon Kim

**Affiliations:** 1 Department of Chemistry, Pukyong National University, Busan 608-737, Korea; E-Mail: ibhatnagar@gmail.com; 2 Marine Bioprocess Research Center, Pukyong National University, Busan 608-737, Korea

**Keywords:** marine antitumor compounds, cancer signaling pathways, shortfalls in cancer research, clinical status of antitumor drugs

## Abstract

Cancer is considered as one of the deadliest diseases in the medical field. Apart from the preventive therapies, it is important to find a curative measure which holds no loopholes and acts accurately and precisely to curb cancer. Over the past few decades, there have been advances in this field and there are many antitumor compounds available on the market, which are of natural as well as synthetic origin. Marine chemotherapy is well recognized nowadays and profound development has been achieved by researchers to deal with different molecular pathways of tumors. However, the marine environment has been less explored for the production of safe and novel antitumor compounds. The reason is a number of shortfalls in this field. Though ample reviews cover the importance and applications of various anticancerous compounds from marine natural products, in the present review, we have tried to bring the current status of antitumor research based on marine inhibitors of cancer signaling pathways. In addition, focus has been placed on the shortfalls and probable strategies in the arena of marine antitumor drug discovery.

## 1. Introduction

Cancer has posed a great challenge to the fields of medicine and immunology. Finding novel and efficient compounds of natural origin has been a major point of concern for research in the pharmaceutical sciences. Nature harbors a vast variety of flora and fauna including microbial inhabitants and lithosphere, atmosphere and hydrosphere cumulatively provide an excellent source of chemically diverse compounds with great therapeutic potential. Drugs derived from natural products have a giant impact on the antitumor drug discovery regime of the present day [1]. This importance of natural products in the field of therapeutics may be attributed to their high affinity to the target, little loss of entropy when they bind to a protein and their bioavailability. Moreover, natural compounds are quite flexible in conformational acquisition in aqueous and lipophilic environments [2].

The oceanic environment has been a vast source of such natural compounds covering a wide range of bioactivity such as photoprotective [3], antihelmintic, antibacterial, anticoagulant, antifungal, anti-inflammatory, antimalarial, antiprotozoal, antituberculosis, antiviral and other miscellaneous mechanisms of action [4]. For the past two decades, marine pharmaceutics has been a developing field in the antitumor drug development arena. Marine organisms including sponges [5,6], sponge-microbe symbiotic association [7], gorgonian [8], actinomycetes [9], and soft coral [10] have been widely explored for potential anticancer agents.

Despite an array of antitumor compounds that have been produced from the marine environment, a vast majority of oceanic habitat is underexplored due to various problems faced by marine biotechnologists and pharmacists. First, the marine natural products are large in molecule size and complex in structure with multiple chiral centers. Second, lack of technological innovations and financial support coupled with environmental considerations pose hindrances to marine antitumor drug research. Some speedy expansions in molecular biology, bioengineering, genomics, proteomics and metabolomics have aided the discovery of natural products and their implication in antitumor drug development.

This review showcases recent developments in the field of marine antitumor research with emphasis on the signal transduction pathways of oncogenesis, clinical status of the marine derived antitumor compounds and discusses the potential challenges and probable strategies to combat the gap between marine compound isolation and successful antitumor drug development. It is an attempt to focus the attention of the scientific community including biologists and chemists on an integrative approach towards antitumor drug development from marine sources

## 2. Marine Drugs against Major Players in Cancer

Carcinogenesis is a complex process controlled by various signal transduction pathways linked to processes such as inflammation, cell differentiation and survival, and metastasis. Most of the players of these pathways are interrelated and irregularities in their crosstalk result in impairment of cellular functions leading to tumor generation and progression. Few such players have been targeted for cancer therapy and some inhibitors from marine sources have been designed and studied to counteract tumor progression and curb carcinogenesis. Some such studies are reviewed in this section.

### 2.1. MMP Inhibitors

Matrix metalloproteinases (MMPs), zinc dependant endopeptidases that degrade the extracellular matrix (ECM) [26], have been extensively focused on due to their evident role in carcinogenesis and cellular invasion by catabolizing the extracellular matrix [27]. Apart from playing a major role in invasion, angiogenesis, and metastasis during tumor progression, MMPs are also important for cancer cell transformation, growth, apoptosis, signal transduction and immune regulation [28,29]. Marine sources have been extensively explored for potential MMP inhibitors [30]. Kim *et al*. (2006) reported the inhibitory effect of marine derived chitooligosaccharides (COS) on the activation and expression of matrix metalloproteinase-2 (MMP-2) in primary human dermal fibroblasts (HDFs). They found that medium molecular weight COS (3–5 kDa) exhibited highest inhibitory effects by reducing the gene expression and transcriptional activity of MMP-2 [31].

Further, Quang *et al*. (2006) investigated the effect of low molecular weight COS (1–3 kDa) on the activity and expression of MMP-9 in HT1080 cells and confirmed the inhibition by gelatin zymography [32]. To understand the molecular basis of the MMP-9 inhibitory effect of COS, Rajapakse *et al*. evaluated carboxylated chitooligosaccharides (CCOS) on human fibrosarcoma cell line. A clear dose-dependent inhibition on MMP-9 mediated gelatinolytic activities were observed in HT1080 cells following the treatment with CCOS in zymography experiments. Transfection studies carried out with MMP-9 and AP-1 reporter constructs suggested that the observed reduction in MMP-9 expression was due to down-regulation of MMP-9 transcription mediated via inhibition of AP-1. However, in the presence of CCOS, NF-κB and TIMP-1 expression levels remained constant [33].

Wang *et al*. (2008) isolated the sulfated *S. maindroni* ink polysaccharide (SIP-SII) from cuttlefish *Sepiella maindroni* and examined the effects of SIP-SII on the expression of matrix metalloproteinases MMP-2 and MMP-9 as well as tumor cell invasion and migration. SIP-SII (0.8–500 mg/mL) significantly decreased the expression of MMP-2 activity in human ovarian carcinoma cells SKOV3. No significant decrease of MMP-9 activity was detected in the cell line after SIP-SII treatment [34]. MMP inhibitory effects of phlorotannins from marine brown algae *Ecklonia cava* (EC) have also been studied. Fluorometric assay revealed that EC extract could specifically inhibit both MMP-2 and MMP-9 activities significantly (P < 0.001) at a concentration of 10 μg/mL in human dermal fibroblasts and HT1080 cells. In addition, EC extract did not exert any cytotoxic effect even at 100 μg/mL, proposing its potential use as a safe MMP inhibitor [35].

### 2.2. HIF Inhibitors

To design effective drugs against cancer, it is mandatory to understand the underlying tumor physiology and the changes occurring in the tumor microenvironment [36]. It has been observed that tumor progression is associated with not only increased microvascular density but also intratumoral hypoxia [37]. Further, loss of HIF-1 activity has been shown to have immense negative effects on tumor growth, vascularization and energy metabolism in xenograft assays [38,39]. Thus a number of HIF inhibitors have been designed with the aim of finding new direction to tumor therapy. Laurenditerpenol, isolated from bioassay-guided fractionation of the lipid extract of the red alga *Laurencia intricata* Lamouroux (Rhodomelaceae), yielded the first marine natural product that inhibited HIF-1 activation [40]. It was shown to inhibit HIF-1 activation by blocking hypoxia-induced HIF-1α protein accumulation and suppressed mitochondrial oxygen consumption at ETC complex I at an IC_50_ value of 0.8 μM.

In search of potent and selective small-molecule HIF-1 inhibitors, Liu *et al*. (2007) observed 81% inhibition of hypoxia-induced HIF-1 activation by the crude extract of the sponge *Dendrilla nigra* (Aplysillidae) at a concentration of 5 μg/mL. The study was carried out using T47D human breast carcinoma cell-based reporter assay and the bioassay-guided chromatographic separation yielded four new lamellarin-like phenolic pyrroles that bear structural features similar to Lamellarin O [41]. A year later, the same group identified and characterized a structurally unique inhibitor of HIF-1 activation, Furospongolide (IC50 2.9 μM, T47D breast tumor cells) from a marine sponge *Lendenfeldia* sp. One new cytotoxic scalarane sesterterpene was also reported from the same extract. They found that Furospongolide blocked the induction of the downstream HIF-1 target secreted vascular endothelial growth factor (VEGF) and suppressed HIF-1 activation by inhibiting the hypoxic induction of HIF-1α protein. It was found to suppress tumor cell respiration via the blockade of NADH-ubiquinone oxidoreductase (complex I)-mediated mitochondrial electron transfer [42].

Lipid extract of the crinoid *Comantheria rotula* (Comasteridae) yielded seven Benzo[*g*]chromen-4- one and Benzo[*h*]chromen-4-one pigments. The crinoid’s pigments significantly inhibited both hypoxia-induced (IC_50_ values 1.7–7.3 μM) and iron chelator-induced HIF-1-dependent luciferase reporter activity (IC_50_ values 0.6 to 3.0 μM) in T47D cells. Four of these pigments were also reported to significantly suppress HIF-1 activation in PC-3 prostate tumor cells [43]. A number of other structurally diverse marine natural product-derived HIF inhibitors including Manassantin B [44], 7-hydroxyneolamellarin A [41] have also been discovered in past some years. An extensive review published last year covers most of the information on HIF inhibitors of natural origin including compounds from marine algae and marine organisms [45].

### 2.3. Nuclear Factor-κB Inhibitors

**Figure 2:**
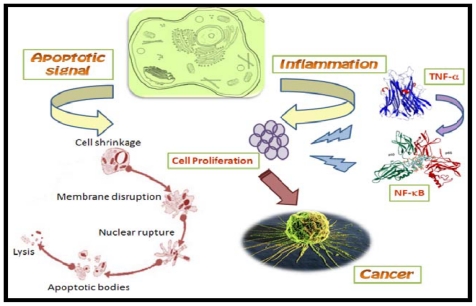
Role of TNF-α induced NF-κB in carcinogenesis. During inflammation, TNF-α induced NF-κB acts on proliferating cells thereby causing their malfunction and leading to cancer.

Nuclear factor-κB (NF-κB) is a ubiquitous transcription factor, a dimer of proteins of the Rel family including NF-κB1 (p50), NF-κB2 (p52), RelA (p65), RelB and c-Rel [46], whose deregulated expression may lead to cancer (Figure 2). NF-κB is activated by various stimuli, including TNF-α (tumor necrosis factor-α), interleukin-1 and lipopolysaccharide [47]. Certain fungal metabolites show promising potential as novel anti-cancer agents as they modulate the activity of NF-κB. Folmer *et al*. (2008) divided NF-κB inhibitors into four categories: (a) compounds targeting the IKK-dependent degradation of IkB, (b) compounds specifically interfering with the proteolytic activity of the 26S proteasome, (c) compounds interfering with the binding of NF-κB to its DNA binding site, and (d) compounds with unknown mechanisms of action [48]. Marizomib (Salinosporamide A; NPI-0052), a proteasome inhibitor isolated from a marine bacterium *Salinispora tropica*, has a great potential as an anticancer drug [49]. A study published last year reported three new cyclohexadepsipeptides, Arenamides (A-C) from the fermentation broth of a marine bacterial strain *Salinispora arenicola*. They studied the effect of Arenamides A and B on NF-κB activity with stably transfected 293/NF-κB-Luc human embryonic kidney cells induced by treatment with tumor necrosis factor (TNF). It was observed that Arenamides A and B blocked TNF-induced activation of NF-κB in a dose- and time-dependent manner [50].

Even marine derived sediments are rich in bioactive substances which may prove to be potential NF-κB inhibitors. Nam and coworkers (2010) have recently isolated Fijiolide A, a potent inhibitor of TNF-α-induced NF-κB activation, from a marine-derived bacterium of the genus *Nocardiopsis*. It was observed to reduce TNF-α-induced NF-κB activation by 70.3%, with an IC_50_ value of 0.57 μM. Their data proposes Fijiolide A as a promising lead for more advanced anticancer testing [51]. The biological effects of Heteronemin, a marine sesterterpene isolated from the sponge *Hyrtios* sp., were studied recently by Schumacher and colleagues on chronic myelogenous leukemia cells. In their observation, heteronemin inhibited both trypsin and chymotrypsin-like proteasome activity at an IC_50_ value of 0.4 μM thereby inhibiting NF-κB activation and proving to be detrimental to cancer cells via apoptosis [52].

### 2.4. Topoisomerase Inhibitors

**Figure 3:**
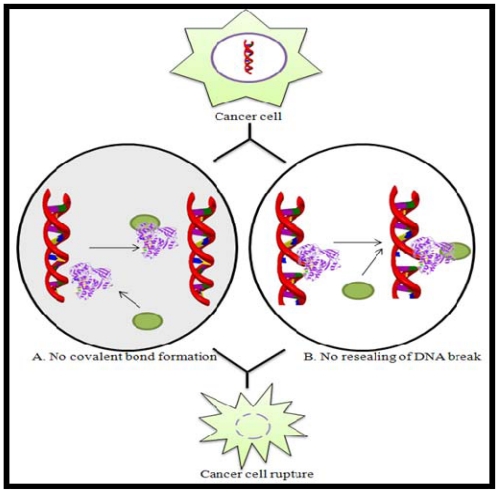
Schematic representation of the mode of action of Topoisomerase inhibitors by (**A**) preventing covalent bond formation or (**B**) preventing DNA resealing. Here, 
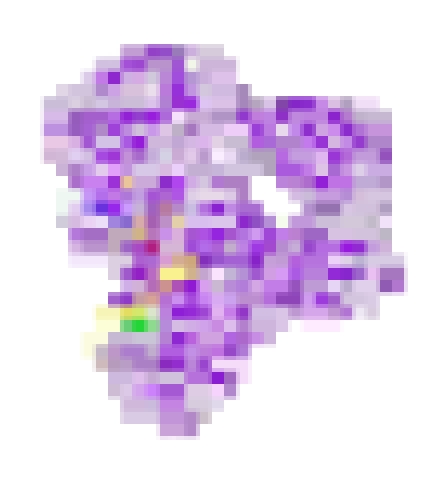
 represents Topoisomerase enzyme and 
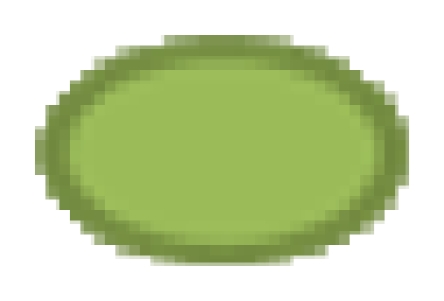
 represents Topoisomerase inhibitor.

Topoisomerases play a major role in maintaining the integrity of the DNA helix during replication, transcription, and chromosome condensation in mitosis [53] and thus are important for cell proliferation. They are now being targeted for anticancer therapy. Over the past three decades, numerous topoisomerase inhibitors have been isolated from various natural sources, in order to find an effective anticancer drug. These agents either prevent the formation of covalent bonds between topoisomerase and DNA or stabilize the intermediate topoisomerase-DNA covalent binary complex thus preventing DNA relegation. They preclude DNA replication and transcription, and thereby lead to the death of cells attempting to undergo these processes (Figure 3).

Makaluvamine A is a pyrroloquinoline, principally isolated from the sponge *Zyzzya fuliginosa* and is known to have potent anticancer activity via inhibiting topoisomerase II [54]. Ascididemin (ASC) is an aromatic alkaloid isolated from the mediterranean ascidian *Cystodytes dellechiajei* [55], which has been shown by Dassonneville and coworkers as a strong inducer of apoptosis in HL-60 and P388 leukemia cells. Through relaxation assays using supercoiled DNA, they showed that ASC stimulated double-stranded cleavage of DNA by topoisomerase II, but exerted only a very weak effect on topoisomerase I [56]. Yet another alkaloid, Lamellarin D (LAM-D), initially isolated from a prosobranch mollusc of the genus *Lamellaria*, has been recently shown to target topoisomerase I. Tardy *et.al.* (2004) conducted studies at the molecular and cellular levels to determine the mechanism of action of this alkaloid and concluded that LAM-D potently stabilizes topoisomerase I–DNA covalent complexes so as to promote the formation of DNA single strand breaks [57].

### 2.5. PKC Inhibitors

Members of the protein kinase C (PKC) family of serine/threonine kinases are known to be major players in oncogenesis. One of the isoforms, PKCɛ, has been demonstrated to increase proliferation, motility, and invasion of fibroblasts or immortalized epithelial cells. It has been proven in xenograft and transgenic animal models that overexpression of PKCɛ is tumorigenic, resulting in metastatic disease [58]. The marine environment has provided a very efficient class of PKC inhibitors, known as Bryostatins. These are highly oxygenated marine macrolides with a unique polyacetate backbone. Since their discovery from the marine animal *Bugula neritina* in 1982 [59], and showing high activity against the murine P388 lymphocytic leukemia, 20 natural Bryostatins are a part of our knowledge today [60]. Their low toxicity and antineoplastic nature makes them promising candidates for cancer chemotherapeutics. Bryostatin-1 is a macrocyclic lactone derived from a marine invertebrate that binds to the regulatory domain of protein kinase C. Short-term exposure to Bryostatin-1 promotes activation of PKC, whereas prolonged exposure promotes significant downregulation of PKC. It has been reported that Bryostatin-1 inhibits proliferation, induces differentiation, and promotes apoptosis in numerous hematological and solid tumor cell lines [61]. It has been proven to be such a promising candidate that it is being evaluated as an antitumor agent against myeloma, acute myeloid leukemia, chronic lymphocytic leukemia (CCL), AIDS-related lymphoma, non-Hodgkin's lymphoma, colorectal, renal, prostate, head and neck, cervix, ovarian, breast, peritoneal, stomach, esophagus, anus and non-small cell lung cancer [60]. Studies are now underway to understand the chemical basis of the biological activity of Bryostatins. Keck *et al.* (2009) prepared a close structural analogue of Bryostatin-1, which differs from it only by the absence of the C (30) carbomethoxy group (on the C (13) enoate of the B-ring), by total synthesis. They further attributed the biological properties of Bryostatin-1 to the substitution in the A-ring [62].

## 3. Clinical Status of Marine Derived Antitumor Drugs

Although the number of natural products is increasing day by day, very few compounds find their way to the market. This may be due to many factors, particularly the cytotoxicity that they show on normal human cell lines, rendering the compound unfit for use as medicinal supplement. Despite the failure rate, some potential compounds have shown promising results as antitumor agents in preclinical trials and many have made it to different phases of clinical trials. Some of them have already been approved as potent anticancer drugs and are already on the market. Cytarabine, Ara-C (Cytosar-U^®^), a DNA polymerase inhibitor is one such drug developed by Bedford laboratories and Enzon pharmaceuticals, which has been approved for use as an antitumor agent. Trabectedin or Ecteinascidin 743 (Yondelis^®^), an alkaloid of tunicate origin, has been approved in European countries to be used for the treatment of soft tissue sarcoma [20]. With an integrated approach of microbiology, screening and natural products chemistry, the marine microbial metabolites are now advancing to be the pharmaceutically important drug candidates. Many compounds of different chemical class and with different mechanisms of action are under clinical trials to prove their potential as antitumor drugs (Table 1).

Eribulin mesylate (E 7389), a macrolide natural product isolated from marine sponges [63] is shown to be a promising microtubule interfering agent leading to cell death by apoptosis [64] and is currently under Phase III clinical trials. Another natural compound under Phase III clinical trials is a peptide Soblidotin (TZT-1027), derived from a marine bacterium [20]. It acts in a dual manner by inhibiting tubulin assembly and disrupting the vasculature of tumor cells, causing them to collapse [65]. Apart from Squalamine lactate, an aminosteroid obtained from shark, and Cemadotin, a peptide obtained from sea slug, few other compounds are under Phase II clinical trials. These include Plitidepsin (Aplidin^®^), a marine depsipeptide from tunicate *Aplidium albicans*, which is a potent apoptosis inducer as depicted in both *in vitro* and *in vivo* studies [66]. Yet another compound is a diketopiperazine known as Plinabulin (NPI-2358), isolated from a marine alga associated *Aspergillus* sp CNC-139 [67]. This compound also inhibits tubulin assembly and acts as a vasculature disrupting agent that destabilizes the tumor vascular endothelial architecture and leads to cell damage [68].

Bryostatin 1, a polyketide isolated initially from Bryozoa, is now known to be produced by its bacterial symbioant, *Candidatus Endobugula sertula* [69]. It basically works as a PKC isozyme inhibitor and is currently under Phase I clinical trial under the aegis of the National Cancer Institute (USA) [20]. Another fungal symbioant, *Penicillium chrysogenum* of the marine sponge *Ircinia fasciculata* produces an antileukemic agent- Sorbicillactone-A, which has qualified now for human trials, owing to its amazing anticancer properties [70]. Marizomib (Salinosporamide A; NPI-0052) Salinosporamide A, a proteasome inhibitor isolated from a marine bacterium *Salinispora tropica* [49], is also undergoing Phase I clinical trials under the auspices of Nereus Pharmaceuticals (San Diego, CA). Interesting properties such as a broader and longer lasting proteasome inhibition, efficacy against a wider range of hematologic malignancies and many solid tumor models, less cytotoxicity to normal cells, higher *in vivo* potency and potential for both oral and intravenous administration makes Salinosporamide A a very promising anticancer agent [71]. Two natural products of cyanobacterial origin (LY355703, CRYPTO52 and Depsipeptide (NSC 630176) are under preclinical trials. Depsipeptide (NSC 630176) is a bicyclic peptide isolated from *Chromobacterium violaceum.* It is known to decrease mRNA expression of the *c-MYC* oncogene thereby causing cell-cycle arrest at G0–G1 [72].

## 4. Shortfalls in Marine Antitumor Drug Research

Although much development has been achieved in the fields of biomedicine and pharmaceutical sciences, still very few natural products have found their way to the market till date. There are ample reasons that may be affecting this shortfall, including; lack of sufficient amount of natural product, difficulties in accessing the source of the samples, problems associated with harvesting of the product, troubles in synthesizing the necessary amounts of the compound, difficulties in isolation and purification procedures, high toxicity of the active compound, ecological considerations, government policies, lack of infrastructure and insufficient capital investment.

For example, studies on the marine HIF inhibitor Laurenditerpenol were hindered by a lack of compound supply [45]. Many species of microorganisms produce specific metabolites at specific phases of their growth. A slight alteration in the culture parameters may lead to changes in the amount and type of metabolite produced thus leading to insufficient supply of the compound. As mentioned earlier in this review, topoisomerase plays a major role in cancer. A new isoform-human topoisomerase III, identified a couple of years ago [73], cleaves single-strand DNA and binds covalently to the 5′-end of the cleaved DNA [74]. Even eight years after its discovery, there are yet no specific inhibitors for topoisomerase III. In addition to this, a recent article in Nature throws light on the dual action of HIF-α in different conditions, which poses a question for its use as a molecular target for cancer therapy [75].

A number of marine microorganisms are difficult to culture and lack of literature on the isolation procedures and standardized culture conditions makes the situation even worse, with less numbers of academics undertaking such studies. For instance, despite the fact that the marine actinomycetes may be a storehouse of novel bioactive molecules, different from terrestrial counterparts, they are an underexploited source for the discovery of novel secondary metabolites [76], the main reason being the lack of efforts spent in exploring them. In addition, the fact that the terrestrial actinomycetes produce resistant spores which get transported from land to oceans, where they can remain available but dormant for many years, has made researchers more skeptical for the existence of indigenous populations of marine actinomycetes [77]. Thus, the myth prevails that marine actinomycetes are originally terrestrial. This lack of knowledge prevents uncovering of the vast potential of marine actinomycetes.

A lack of understanding of cancer biomarkers (major players in carcinogenesis and tumor progression) is also a major shortfall in harnessing the marine environment for specific tumor targeting drugs. Despite a vast array of technologies in cancer research with unprecedented depths of analyses, the complexity of cancer proteomics and the underlying altered signaling pathways pose a great challenge for marine antitumor drug research. There is an imperative need for extensive, integrative and collaborative endeavors to explicate proteome alterations in cancer [78]. Moreover, even after knowing the targets and the development of novel marine drugs against the same, fewer and fewer breakthrough ideas find their way out of research institutions and into the hands of experienced clinicians and medical product development teams as the promising research and innovation are not being translated into new treatments. The increasing drug resistant mutations in cancer causing microbial agents, further leads to the lesser number of antitumor drugs in the market.

Certain other issues that pose an obstacle to the marine antitumor drug research include lack of funding and infrastructure resources and experience in the biotechnology firms to perform the extensive late-stage clinical development programs that are needed for regulatory approval of a drug. The reluctance of large pharmaceutical companies to invest in early-stage research raises another financial setback for marine natural product research. It may be because of the lack of surety of novelty and proven market potential; moreover, it even requires a longer period of time for the investments to be actually returned in terms of profit. The huge structural diversity of marine natural compounds makes it difficult for the isolation and purification of these compounds and the development of novel antitumor drugs from these natural sources possesses problems that are not usually met when one deals with synthetic compounds.

Prevailing threats to global marine biodiversity including overfishing, habitat loss, invasive species and pollution, rising water temperatures and ocean acidification are further making marine antitumor drug research more and more difficult.

## 5. Strategies to Combat the Pitfalls

The discovery of modern anticancer drugs aims to search for improved cytotoxic agents in terms of the accuracy, efficacy and target specificity and sensitivity. The vast biodiversity of the marine environment can serve as a rich source of such natural compounds. However, keeping the above mentioned challenges in view, it becomes mandatory to design specific strategies for improvement of their therapeutic potential.

A semisynthetic approach could be one of the alternatives to enhance the yield of lead natural compounds. It can be achieved by modifying the functional groups of existing natural compounds. This would lead to the generation of structural analogues with greater pharmacological activity and with fewer side effects. For example, studies on the marine HIF inhibitor Laurenditerpenol were hindered by a lack of compound supply. However, a recently completed total synthesis has resolved the absolute configuration of Laurenditerpenol [79] and together with other synthetic efforts [80] may provide sufficient compound to allow further evaluation. A more integrated approach comprising high-throughput screening methods, computational biology and bioinformatics may be useful in producing compounds that are more efficient than the ones that are prevalent today. Increasing use of genomics and implication of combinatorial biosynthesis can be applied for discovery and modification of natural marine microbial products.

Marine fungi are prolific producers of bioactive compounds including antitumor drugs [81–83]. A more intrinsic insight in the biology and interspecies relationship of marine fungi would help for better understanding of the kind of compounds that may be isolated from them. As per Raghukumar (2008), a more systematic research should be initiated for fungi from deep-sea, hypoxic zones (with low oxygen levels) and hydrothermal vents for enzymes, degradation of xenobiotics and bioremediation applications. He further suggested that genomic and proteomic studies with novel organisms such as *Corallochytrium limacisporum* as a model of animal fungal allies will hopefully help in basic research on evolutionary biology [84]. Macrofungi has already been a great source of anticancer agents [85]. An evolutionary approach coupled with an ecological perspective can do wonders in the field of marine natural product discovery and a careful approach in this field can lead to increased production of bioactive compounds with high efficiency. Another effective strategy would be molecular modification for example, implication of ribosome engineering to develop mutant strains that overproduce secondary metabolites by screening various drug-resistance mutations. The efficacy of the strain improvement has recently been highlighted in *Streptomyces* sp. by introducing combinatory drug-resistances [86].

Targeted therapy has been an indispensable approach towards oncogenic signaling pathways. Drug-specific modulation of oncogenic signaling pathways in specific patient subpopulations can predict responsiveness to targeted therapy. Andersen *et al.* (2010) recently reported a pathway-based phosphoprofiling approach to identify and quantify clinically relevant, drug-specific biomarkers for phosphatidylinositol 3-kinase (PI3K) pathway inhibitors that target AKT, phosphoinositide-dependent kinase 1 (PDK1), and PI3K–mammalian target of rapamycin (mTOR) [87]. Another alternative would be to improvise different targeted cancer proteome projects and initiatives with clearly delineated objectives. Advances in the field of proteomics, genomics and metabolomics are integral to cell metabolism and have great impact on the identification of new antitumor targets. Not only this, there are newer fields of biology, such as pharmacogenomics and pharmacogenetics, which are proving to be very helpful for understanding patient susceptibility to specific pharmacological agents.

As far as the problems associated with HIF are concerned, Semenza (2002) suggested that the combination of an anti-angiogenic agent and an inhibitor of HIF-1 might be particularly efficacious, as the angiogenesis inhibitor would cut off the tumor’s blood supply and the HIF-1 inhibitor would prevent the ability of the tumor to adapt to the ensuing hypoxia and thus the efficacy of HIF inhibitor may be greatly enhanced [36]. As suggested by Borkakoti (2004), the use of MMP inhibitors in combination therapy and in early stages of disease onset, could be a novel approach geared towards achieving improved clinical advantage [88].

## 6. Conclusions

One of the greatest challenges for the field of medicine and immunology is finding a sure shot cure for cancer. An extensive research regime needs to be started to combat the ability of cancer cells to mutate and become resistant to available drugs. As mentioned above, collaborative scientific approaches with a focus on molecular mechanisms of tumorigenesis need to be initiated. Efforts are required to gain deeper knowledge regarding the various signal transduction pathways linked to cellular processes such as inflammation, cell differentiation and survival, carcinogenesis, and metastasis. Although it is very difficult to predict the final outcome of such a scheme of drug discovery, it can be assured that a focused approach and combined efforts would definitely accelerate the development of new marine antitumor drugs to be discovered with increased efficiency.

## Figures and Tables

**Figure 1:**
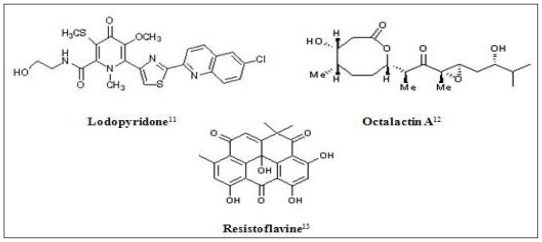

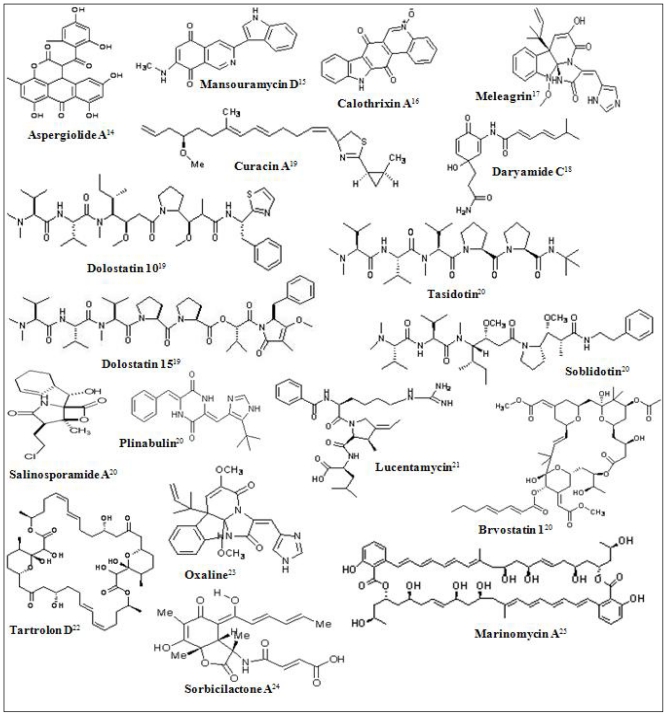
Chemical structures of some marine derived antitumor compounds. The corresponding references have been superscripted after the compound name [11–25].

**Table 1:** Clinical status of marine derived antitumor agents, their chemical class and mode of action.

Compound Name	Chemical Class	Organism	Mode of action	Company	Status
**Cytarabine, Ara-C**	Nucleoside	Sponge	DNA Polymerase Inhibitor	Bedford, Enzon	Approved
**Trabectedin (ET-743)**	Alkaloid	Tunicate	Cell cycle arrest	PharmaMar	Approved
**Eribulin Mesylate (E7389)**	Macrolide	Sponge	Microtubule interfering agent	Eisai Inc.	Phase III
**Soblidotin (TZT 1027)**	Peptide	Bacterium	Microtubule interfering and vascular disrupting agent	Aska Pharmaceuticals	Phase III
**Squalamine lactate**	Aminosteroid	Shark	Calcium binding protein antagonist	Genaera	Phase II
**Cemadotin**	Peptide	Sea slug	Microtubule interfering agent	Knoll	Phase II
**Plinabulin (NPI-2358)**	Diketopiperazine	Fungus	Vascular disrupting agent	Nereus Pharmaceuticals	Phase II
**Plitidepsin**	Depsipeptide	Tunicate	Apoptosis inducer	PharmaMar	Phase II
**Elisidepsin**	Depsipeptide	Mollusc	-	PharmaMar	Phase II
**Zalypsis**	Alkaloid	Nudibranch	Cell cycle arrest	PharmaMar	Phase II
**Tasidotin, Synthadotin (ILX-651)**	Peptide	Bacterium	Microtubule interfering agent	Genzyme Corporation	Phase II
**Discodermolide**	Polyketide	Sponge	Microtubule interfering agent	Novartis	Phase I
**HT1286**	Dipeptide	Sponge	Microtubule interfering agent	Wyeth	Phase I
**LAF389**	Amino acid derivative	Sponge	Methionine aminopeptidase inhibitor	Novartis	Phase I
**Kahalalide F**	Cyclic depsipeptide	Sea slug/alga	Lysosomotropic	PharmaMar	Phase I
**KRN7000**	α-galactosylceramide	Sponge	Immunostimulatory	Kirin	Phase I
**Bryostatin 1**	Polyketide	Bacterium/Bryozoa	PKC isozyme inhibitor	National Cancer Institute	Phase I
**Hemiasterlin (E7974)**	Tripeptide	Sponge	Microtubule interfering agent	Eisai Inc.	Phase I
**Marizomib, Salinosporamide A; NPI-0052)**	Beta-lactone-gamma lactam	Bacterium	Proteasome inhibitor	Nereus Pharmaceuticals	Phase I
**LY355703, CRYPTO 52**	Cryptophycin	Cyanobacterium	Microtubule interfering agent	-	Preclinical
**Depsipeptide (NSC 630176)**	Bicyclic peptide	Cyanobacterium	Histone deacetylase inhibitor	-	Preclinical
